# Evaluation of Sacroiliac Joint Shape in Women with Ankylosing Spondylitis According to Mode of Birth Delivery: A Retrospective Study

**DOI:** 10.3390/medicina61010039

**Published:** 2024-12-30

**Authors:** Sakine Rahimli Ocakoglu, Alper Vatansever, Zeliha Atak, Nurefsan Yanardag, Belkis Nihan Coskun, Sinem Akselim, Gokhan Ocakoglu

**Affiliations:** 1 Department of Obstetrics and Gynecology, Bursa City Hospital, 16110 Bursa, Turkey; 2 Department of Anatomy, Faculty of Medicine, Bursa Uludag University, 16059 Bursa, Turkey; 3 Department of Rheumatology, Faculty of Medicine, Uludag University, 16059 Bursa, Turkey; 4 Department of Physical Medicine and Rehabilitation, Bursa City Hospital, 16110 Bursa, Turkey; 5 Department of Biostatistics, Faculty of Medicine, Bursa Uludag University, 16059 Bursa, Turkey

**Keywords:** ankylosing spondylitis, statistical shape analysis, sacroiliac joint, caesarian section, vaginal delivery, anatomical changes

## Abstract

*Background and Objectives*: Ankylosing spondylitis (AS) is a chronic progressive inflammatory process of the axial skeleton and sacroiliac joints (SIJ). Symptoms typically appear between the ages of 20 and 40, although there are also cases of juvenile-onset AS. This suggests that most patients with AS are of reproductive age at the time of diagnosis. The study aimed to identify differences in the shape of the sacroiliac joint depending on the type of birth (vaginal delivery (V/D) and the cesarean section(C/S) in patients with ankylosing spondylitis. *Materials and Methods*: On pelvis X-ray images of women n = 36 with AS and n = 34 in the control group, 12 landmarks were marked, and differences in SIJ shape between the study groups were assessed using generalized Procrustes Analysis. *Results*: The results showed that the anterior point of the SIJ had an enlarged shape in the V/D group compared with the C/S group, even in the control group. There was a difference between the mean right and left SIJ shapes of the AS group patients with V/D and the controls with C/S (*p* = 0.007 and *p* < 0.001). The superior part of the right SIJ tended to be enlarged in V/D-delivered AS patients, compared to the C/S control group. On the left side, the middle region of the SIJ was statistically enlarged in AS patients with V/D compared to the healthy C/S group. *Conclusions*: This study demonstrates that vaginal delivery is associated with increased sacroiliac joint (SIJ) enlargement in both healthy individuals and those with ankylosing spondylitis (AS). Our findings suggest that delivery type independently influences SIJ morphology, and cesarean section (C/S) may serve as a protective procedure for preserving SIJ shape in AS patients. These results underline the importance of individualized obstetric planning for AS patients to mitigate potential risks to SIJ morphology.

## 1. Introduction

Ankylosing spondylitis (AS) is known as a chronic inflammatory disease characterized by inflammation of the axial skeleton [1,2]. The prevalence of AS worldwide is between 0.1 and 1.4%, which is thought to be more common in people with low socioeconomic conditions [3].

Research shows that AS has existed since ancient times. Excavations of mummies found in ancient Egypt showed that the pharaoh also suffered from this disease [4]. Many scientists have tried to describe this pathological condition before and after the common era. Formerly, the diagnosis of AS was based on clinical symptoms, such as numbness, joint pain and swelling, typical spinal posture, and inflexibility; however, AS is recently known as a seronegative spondyloarthropathy, meaning that tests show no presence of rheumatoid factor antibodies with radiographic-revealed axial spondylitis [5]. AS primarily affects the spine and sacroiliac joint (SIJ) and is characterized by inflammation-causing ankylosis [1,2]. Ankylosis occurs due to the ossification of the vertebral ligaments and SIJ (attachment point of the spine to the sacrum bone), causing fusion of the vertebral bodies and leading to the final immobilization of the intervertebral and SI joints. Symptoms of AS typically start during the late teens and early twenties [6]. Clinical symptoms of AS are varied and include ossification-induced changes (lumbar back pain, limitation of mobility and stiffness, limitation of chest expansion) and other system-involvement symptoms (uveitis, inflammatory bowel disease, psoriasis, and aortitis).

### 1.1. Sacroiliac Joints and AS

If the SIJ is involved in the inflammatory process of AS, this condition may be referred to as pelvospondylitis [7]. Characteristic findings on conventional radiography (X-rays) and/or magnetic resonance imaging (MRI) are key to diagnosis. In addition, radiological assessment with X-rays or MRIs is a useful tool for monitoring the disease. Erosion and ankylosis of the sacroiliac joints are observed radiographically in AS [8].

**Figure 1:**
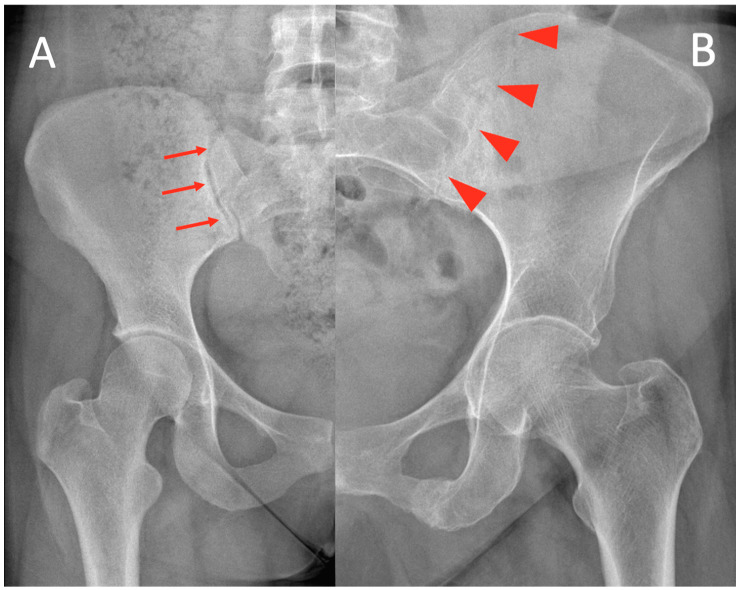
(**A**) A healthy sacroiliac joint is demonstrated with red arrows; (**B**) A sacroiliac joint with AS; red arrowheads show enlargement due to ossification.

Anatomical changes in sacroiliitis are usually bilateral and symmetrical and are often the first alteration of AS. A radiographic diagnosis of sacroiliitis is a prerequisite, and the presence of one or more clinical features is essential for a definite diagnosis of AS [9]. Early radiographic changes predominate on the iliac side of the cartilage compartment. As a result of the erosion of the subchondral bone, a loss of definition of the articular surfaces occurs, usually accompanied by varying degrees of osteoporosis of the adjacent areas and surrounding reactive sclerosis. Bone erosion causes radiographic observations of focal widening of the joint space (Figure 1).

The sacroiliac joint (SIJ) exhibits significant anatomical disparities between healthy persons and those afflicted with ankylosing spondylitis (AS), frequently affected by abnormal joint morphology. Individuals with AS exhibit a higher prevalence of atypical joint morphologies, including intraarticular variations and crescent-shaped joints. These unusual forms are associated with an increased probability of erosions and bone marrow edema (BME) [10]. Comparable changes are also more prevalent in individuals with mechanical joint disease (MJD) and axial spondyloarthritis (axSpA) than in healthy controls. Investigations into sacroiliac joint morphology indicate the presence of both articular and extra-articular components, with the latter being misidentified as the joint itself. This can make it more challenging to assess degenerative or inflammatory disorders [11]. Sacroiliitis patterns vary between primary and secondary axial spondyloarthritis. Individuals with primary axSpA typically exhibit more symmetrical sacroiliitis and extensive spinal bone marrow edema [12]. These findings underscore the significance of acknowledging variations in joint morphology and their contribution to diagnosing and comprehending AS and associated disorders.

As the disease progresses, the SIJ loses its definition, with a radiological overlay of erosions, sclerosis, and ossification filling the erosions and the original cartilaginous “joint space.” In the later stages of the disease, the joint may disappear completely with ankylosis and bone remodeling [13].

### 1.2. Sacroiliac Joint and Pregnancy

Changes in the SIJ in healthy women during pregnancy occur due to various biomechanical mechanisms, such as weight gain, posture (exaggerated lordosis), intra-abdominal pressure, and laxity of the spine and pelvic structures [14]. The ligaments of the sacroiliac joint are the anterior sacroiliac, interosseous sacroiliac, posterior sacroiliac, and extrinsic sacroiliac joint ligaments. During pregnancy, the production of relaxin, a hormone involved in weakening ligaments and the pubic symphysis, increases. This ensures a wide opening of the pelvic joint during labor [15].

Women with ankylosing spondylitis (AS) undergo substantial alterations in the sacroiliac joint (SIJ) throughout pregnancy and postpartum. These alterations are affected by a confluence of physical stress, hormonal variations, and the impact of the disease itself. Studies indicate that bone marrow edema (BME) frequently occurs during pregnancy, with a tendency to reach its zenith postpartum. By three months after giving birth, about 69% of women show signs of bone marrow edema (BME), and around 41% meet the criteria for sacroiliitis within a year [16,17]. Interestingly, for women with early-stage axial spondyloarthritis, pregnancy does not seem to significantly worsen sacroiliac joint abnormalities on imaging, suggesting that it may not have a significant impact on the condition [18]. The postpartum period brings significant challenges, with 45% of women experiencing a flare-up of AS symptoms after giving birth [19]. These findings emphasize the importance of carefully interpreting imaging results during the postpartum period since some changes may mimic or hide true disease activity [16,17].

With the integration of advanced imaging technology and software (radiological imaging methods) into medicine, geometric changes in the shape of an organ or structure can be analyzed using anatomically significant key points, also called landmarks, which have anatomical significance. By taking the coordinates of these landmarks as variables, it is possible to analyze geometric shapes based on measurements taken from organisms. Statistical shape analysis, a modern geometric morphometric method, uses organ or organism shapes as input data [20]. It is an auxiliary tool for interpreting structural differentiation and deformations in the relevant organ by considering shape changes. Numerous studies in medicine consider the organ or organism under examination regarding its geometrical properties [21,22,23].

AS is attracting the attention of many scientists and has been the subject of extensive research. The first precise clinical descriptions appeared in the last decades of the 18th century [24]. Changes in SIJ morphology are well-documented in AS; however, the potential impact of delivery type on SIJ shape remains underexplored. To date, there is no consensus regarding the preferred type of delivery for patients with AS. A study showed that pregnancy and VD may not be factors associated with the worsening of AS [25]. The current literature indicates that sacroiliac joint (SIJ) pain can occur postpartum, even in patients without an ankylosing spondylitis (AS) diagnosis. Such postpartum changes may mimic sacroiliitis and highlight the role of delivery type in influencing SIJ morphology [18]. On the other hand, one study conducted with AS-diagnosed women aged 19–49 years showed that pregnancy and delivery have no effect on the radiographic progression of spine and sacroiliac joints in women with AS [26].

However, no studies to date compare SIJ shape differences in AS and normal patients based on delivery type using geometric morphometric analysis. This study seeks to address this gap by evaluating SIJ morphometry in patients with AS and healthy controls who delivered via vaginal delivery (V/D) or cesarean section (C/S). The findings aim to guide future research and clinical practice, particularly regarding delivery mode selection in AS patients.

## 2. Materials and Methods

This study utilized the anteroposterior pelvic X-ray images of 70 women, n = 36, with ankylosing spondylitis and n = 34, for the control group.

Radiographic grading of X-rays for both right and left sacroiliac joints (SIJ) in patients with ankylosing spondylitis (AS) was performed by a rheumatologist based on the radiographic criteria for Modified New York Stages. Additionally, the suitability of the images for the study was evaluated by an anatomist to ensure accuracy and consistency. Radiological evaluations for the present study were conducted after delivery (in post-delivered patients) with varying time intervals between delivery and the X-ray examination. This variability reflects the study’s retrospective design and was inherent to the study population and clinical practice. The X-ray images of patients in the study group diagnosed with AS and the control group were evaluated retrospectively between November 2021 and February 2023 at Bursa City Hospital.

### 2.1. Study Design and Setting

This retrospective study included patients diagnosed with ankylosing spondylitis (AS) at the rheumatology clinic of Bursa City Hospital, which has been actively operating for six years. All patients were diagnosed within the past five years, and their obstetric history, including delivery type, was collected retrospectively from medical records.

1. Inclusion Criteria for the Study Group:

- Were diagnosed with AS based on clinical and radiological criteria;
- Being between the ages of 18–60;
- Being diagnosed with ankylosing spondylitis at Bursa City Hospital;
- Having an anteroposterior pelvic X-ray available for analysis;
- No pelvic trauma or fracture in the anamnesis’
- Data accessibility.

2. Inclusion Criteria for the Control Group:

- Being between the ages of 18–60;
- Having an anteroposterior pelvic X-ray for other indications;
- No pelvic trauma or fracture in the anamnesis;
- Data accessibility.

3. Exclusion Criteria from the Study:

- Incomplete medical records;
- Patients diagnosed with ankylosing spondylitis in our hospital but underwent other radiological examinations (magnetic resonance imaging or ultrasound);
- Pelvic trauma or fracture in the anamnesis;
- Data inaccessibility.

This study used the Siemens Multix Fusion Digital Radiography System. The patient holds his breath and remains still in the supine position, and pelvic radiographs are taken with an X-ray dose of (75–80) kVp-58 mAs. The X-ray Image sections were collected after anonymizing participants’ age(year), obstetric data type of delivery vaginal(V/D) or cesarean section (C/S), gravida, parity, and abortus. The Bursa City Hospital ethics committee approved this prospective study and the study protocol (No: 2023-10/9).

### 2.2. Collection of Two-Dimensional SIJ Landmarks

**Figure 2:**
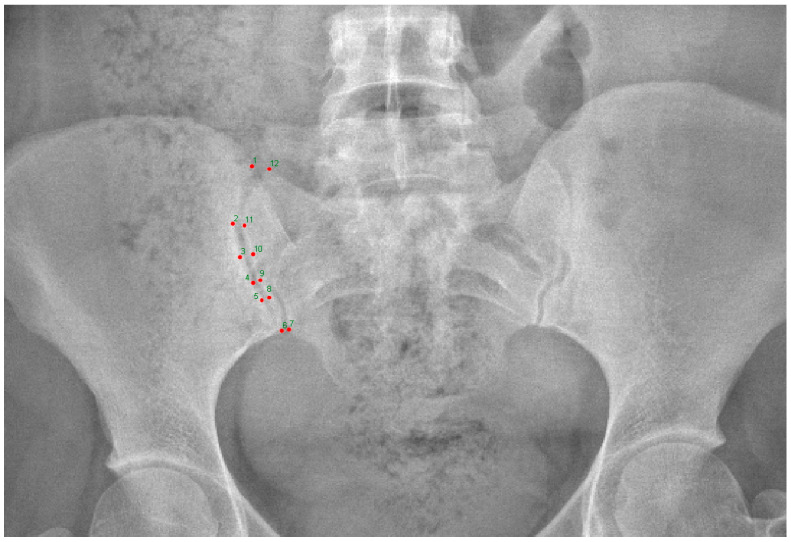
Landmark markings on the sacroiliac joint.

Data on the regions marked in the sacroiliac joints (right-left sides) were collected from two-dimensional digital images. Twelve landmarks of SIJ were considered from the image corresponding to the anterior–posterior X-ray images (Figure 2) and marked using TPSDIG version 2.04 software. The landmarks were selected based on reliability and anatomical coverage to provide the most comprehensive explanations of SIJ morphometry. The identifications of these markings are presented in Table 1.

### 2.3. Statistical and Geometric Morphometric Analysis

Centroid size measurements, which is one of the numerical quantities obtained as a result of the comparison of the right sacroiliac joint shapes of the patients with ankylosing spondylitis who gave birth with normal vaginal delivery and the control group who gave birth with normal vaginal delivery, which is among the main findings of our study, were used for posthoc power analysis.

Centroid size, commonly used in geometric morphometrics as a measure of size, is calculated as the square root of the sum of the squared distances from all landmarks of an object to their centroid. The centroid’s position is determined by averaging all landmarks’ x and y coordinates [27].

The mean centroid size measurement of the patients in the AS group who delivered by normal vaginal delivery was calculated as 228.90 ± 42.88, and the mean centroid size measurement of the patients in the control group who delivered by normal vaginal delivery was calculated as 135.61 ± 15.15. The effect size value was calculated as d = 2.90 based on the groups’ mean and standard deviation values. Using the calculated effect size value, type I error was fixed at 5%, the allocation ratio value was taken as 0.89, and the study’s power was calculated as >95% due to the post hoc power analysis conducted in light of the current findings. Posthoc power analysis was performed using GPower software [28].

The Shapiro–Wilk test was used to analyze the conformity of the age variable to the normal distribution. Age was expressed as mean and standard deviation and compared between study groups using independent samples t-test. Gravida and parity were expressed as median, minimum, and maximum values and compared between study groups using the Mann–Whitney U test. Type of birth and abortion rates were compared between groups using the Chi-square test and Fisher–Freeman–Halton test.

The shape difference of sacroiliac shapes between study groups was assessed by performing a Generalized Procrustes Analysis. Box’s M procedure tests the equality of variance–covariance matrices. The James F_J_ test, based on a resampling procedure, was considered for shape comparisons [20].

In this study, R 3.5.1 [29], PAST 3 [30], and SPSS (Version 21.0. Armonk, NY: IBM Corp.) software were used for statistical analysis.

### 2.4. Landmark Reliability

The generalizability theorem (GT) was employed to assess the interrater reliability coefficient for a 2-facet crossed design (landmark pairs by rater and by subject) [31]. The generalizability coefficient is utilized in the GT to describe the reliability of relative (norm-referenced) interpretations. In this research, a single rater identified the anatomical landmarks. The rater’s reliability was evaluated using repeated landmarks. A single investigator collected landmarks on the SIJ, and after a month, the same investigator re-identified the same landmarks on the same 20 subjects (10 cases and 10 controls). The study revealed that (G = 0.9951) has good repeatability. The landmark reliability was calculated using the following web link: http://biostat.home.uludag.edu.tr/landmark_reliability/G_coefficient.html accessed on 27 December 2024 (Bursa, Turkiye).

## 3. Results

Comparisons between patients with AS and controls according to demographic characteristics are given in Table 2. The mean age of patients in the control group was lower than in patients with ankylosing spondylitis (38.70 years vs. 46.75 years; *p* < 0.001). No significant differences were observed between the groups with regard to the mode of delivery (*p* = 0.469). Furthermore, the groups had no differences in gravida and parity (*p* = 0.218 and *p* = 0.270, respectively). Similarly, abortion rates did not vary significantly between the groups (*p* = 0.684).

The radiologic evaluation of sacroiliac joints (SIJ) in the ankylosing spondylitis (AS) group (Table 3), based on the Modified New York Criteria, did not show any significant differences in staging between delivery types (C/S vs. V/D) for the right SIJ (*p* = 0.862) or the left SIJ (*p* = 0.413).

**Figure 3:**
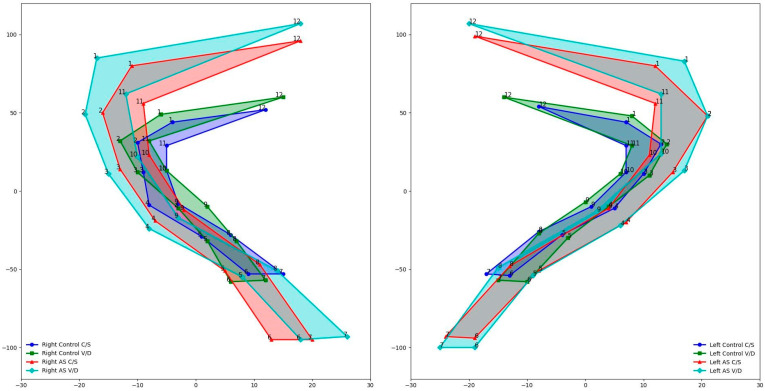
Procrustes mean shapes of sacroiliac joints of controls and ankylosing spondylitis patients according to type of birth.

The Procrustes mean shapes of the right and left sacroiliac joints of patients with ankylosing spondylitis and the control group according to the type of birth are given in Figure 3.

**Figure 4:**
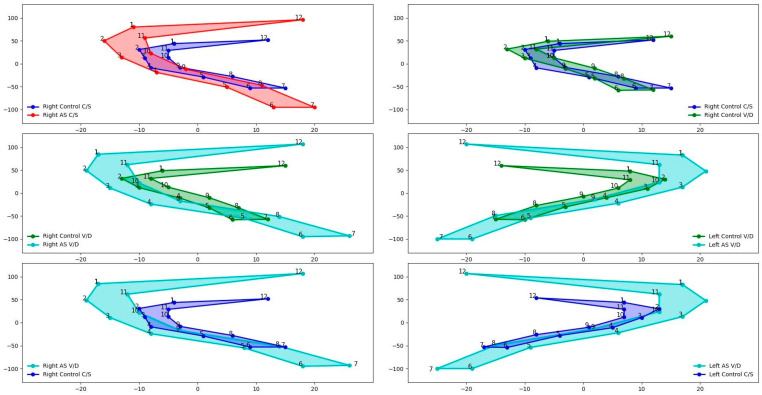
Significant differences in the Procrustes mean shapes of sacroiliac joints.

As a result of the analyses for comparing sacroiliac joint shape, the significant comparisons are shown in Figure 4.

**Figure 5:**
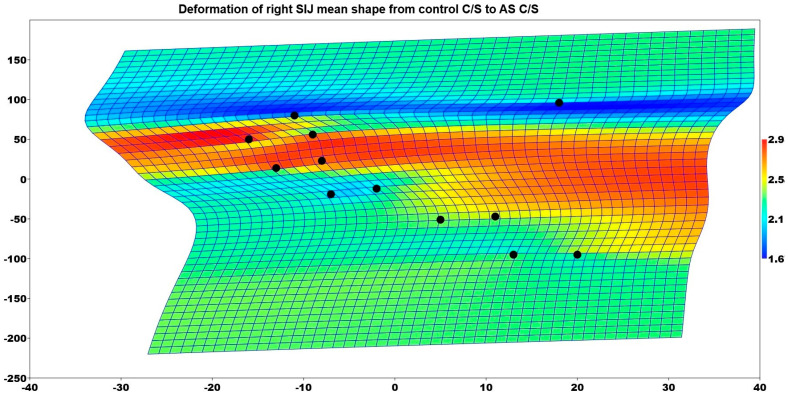
Deformation of the right SIJ mean shape from control C/S to AS C/S.

It was determined that the right SIJ shape of the patients who gave birth by C/S differed between the control and AS groups (*p* = 0.004). Figure 5 illustrates the deformation of the mean shape of the right SIJ in comparison to the mean shape of the control group patients who had C/S deliveries and the AS group patients who had C/S deliveries. The enlargement in the SIJ for AS patients was especially seen in the upper part of the joint.

**Figure 6:**
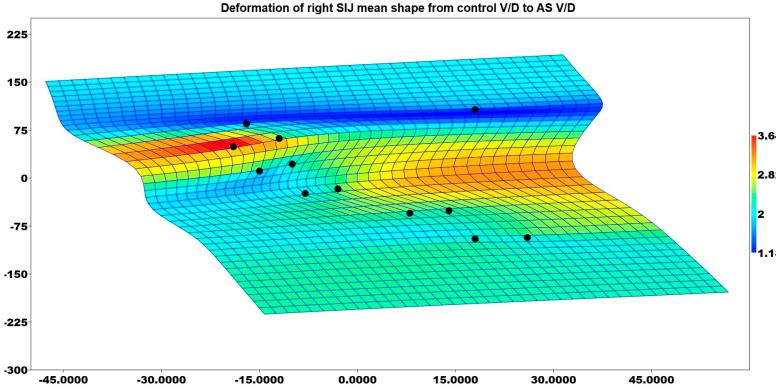
Deformation of right SIJ mean shape from control V/D to AS V/D.

The study revealed that the right SIJ shape of patients who gave birth vaginally differed between the control and AS groups (*p* = 0.004). Figure 6 illustrates the deformation of the mean shape of the right sacroiliac in comparison to the mean shape of the control group patients who had V/D deliveries and the AS group patients who had V/D deliveries. Although the surface area was not as high as in the AS C/S group, the AS V/D group had enlarged SIJ at the upper part of the joint.

**Figure 7:**
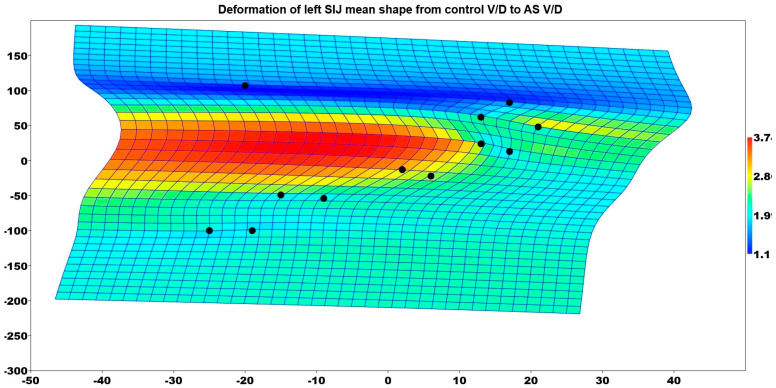
Deformation of left SIJ mean shape from control V/D to AS V/D.

Similarly, the left SIJ shape of these patients also differed between the two groups (*p* = 0.018). Figure 7 illustrates the deformation of the mean shape of the left SIJ in comparison to the mean shape of the control group patients who had V/D deliveries and the AS group patients who had V/D deliveries. The point between the upper 1/3 and middle 1/3 part of the SIJ had a significantly enlarged shape.

**Figure 8:**
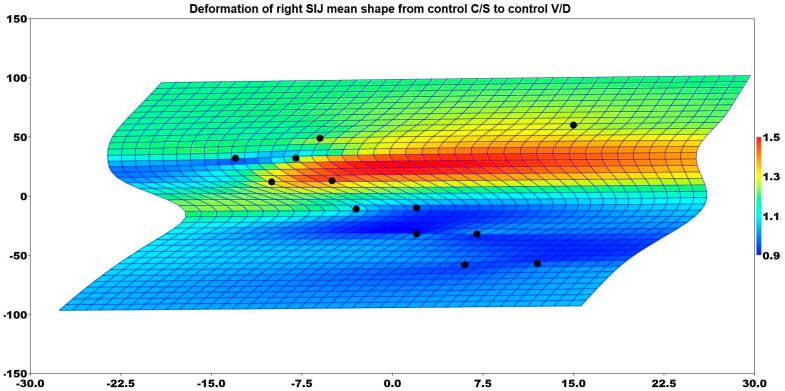
Deformation of right SIJ mean shape from control C/S to control V/D.

The control group also demonstrated a significant difference in the right SIJ shape between patients with a C/S and those with a V/D (*p* = 0.039). Figure 8 illustrates the deformation of the mean shape of the right sacroiliac in comparison to the mean shape of the control group patients who had C/S deliveries and the control group patients who had V/D deliveries. According to anatomical landmarks, the most anterior point of the SIJ had an enlarged shape in the V/D group compared to the C/S group in control cases.

**Figure 9:**
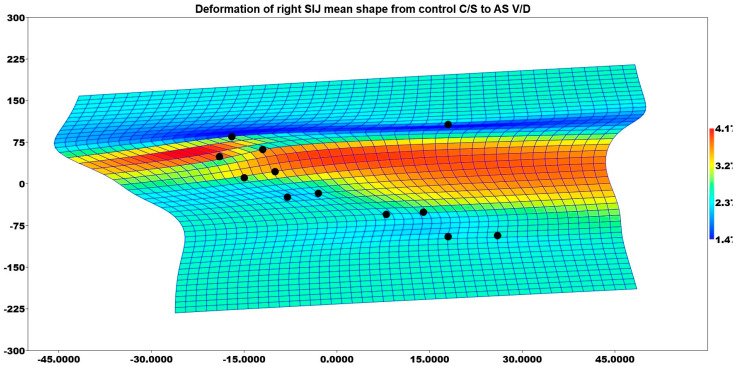
Deformation of right SIJ means shape from control C/S to AS V/D.

There was a difference between the mean right and left SIJ shapes of the AS group patients with V/D and the controls with C/S (*p* = 0.007 and *p* < 0.001). Figure 9 illustrates the deformation of the mean shape of the right SIJ in comparison with AS-V/D to control the C/S group patients. The superior part of the SIJ tended to be enlarged in AS with V/D on the right side.

**Figure 10:**
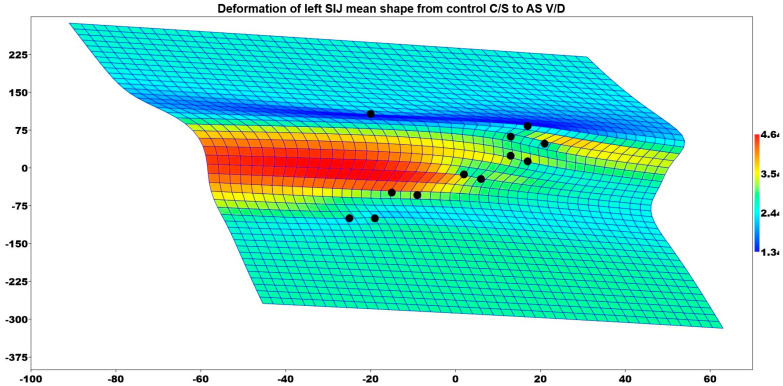
Deformation of left SIJ means shape from control C/S to AS V/D.

Figure 10 illustrates the deformation of the mean shape of the left sacroiliac in comparison to the mean shape of the control C/S group patients. On the left side, the middle region of the SIJ was statistically enlarged in AS patients who had V/D compared to the healthy and C/S groups.

## 4. Discussion

According to our knowledge, no studies have utilized geometric morphometric analysis to compare SIJ shape differences in AS and normal patients based on delivery type. This study assessed the impact of AS on the morphometric characteristics of the SIJ, specifically in relation to two types of delivery: V/D versus C/S.

In most cases, AS symptoms begin between the ages of 20 and 40, although there are also patients diagnosed with AS under the age of ≤16 years (juvenile-onset ankylosing) [32]. According to the source of Turkiye data—TurkStat, the mean age of the parity was 26.7 in 2001 and 29.0 in 2020. The mean age of the primiparous in 2020 was 26.5. This indicates that most patients with ankylosing spondylitis are in their reproductive years at diagnosis [33]. The emergence of the disease in the fertile age makes AS women a vulnerable pregnant group and requires an individual approach during pregnancy and the peripartum period.

Known risk factors for SIJ-related pain include obesity, mild trauma caused by jogging, lumbar spinal fusion, scoliosis, leg length discrepancy, gait abnormalities, and also pregnancy [34]. The SIJ is the largest axial joint in the body. The auricular surfaces of the sacrum and ilium bones are separated by a joint space (0.5–4 mm) containing synovial fluid and surrounded by a fibrous capsule [35]. As a person becomes older, the space between joints shrinks and becomes less regular. Additionally, it is important to note that joint fusion does not occur during aging [36]. In our study, the mean age was higher in the AS group. However, the age difference between the study groups, as shown in studies, is not a factor affecting the changes in SIJ [36]. On the other hand, the results of our research showed that the delivery type alters the SIJ’s shape. AS patients delivered by V/D had significantly increased SIJ due to ossification compared to patients delivered by C/S. Interestingly, the enlargement was more noticeable, especially on the right side.

During pregnancy, the production of relaxin, a hormone involved in weakening ligaments of SIJ and the pubic symphysis, increases. This ensures a wide opening of the pelvic joint during delivery [15]. Adaptively, compared to men, women have more mobility of the SIJ since it has an increased pubic angle and a decreased curvature of the SIJ. These differences evolutionarily facilitate the birth process, with the participation of hormones such as relaxin and estrogen, which cause symphysiolysis. While these changes may be necessary for delivery, they also cause an increased risk of pelvic pain [37]. Pregnancy-induced back pain is considered a typical physiological symptom of pregnancy due to its extremely high prevalence [38]. It should be noted that conditions such as weight gain, lordosis, and birth injuries may cause joint bleeding during delivery and subsequently increase the risk of sacroiliitis [39]. Godke et al. showed that 26% of women had SIJ dysfunction in the postpartum period. The highest prevalence was observed in vaginal term deliveries compared to cesarean sections [40].

However, there is no consensus in the current literature regarding the effects of AS on pregnancy outcomes and the preferred type of delivery for AS patients. Moreover, the relationship between AS and specific complications during pregnancy, the delivery process, and fetal health is not defined yet [41]. Some research demonstrated that the AS caused an increased C/S delivery rate [42,43]. Research reported that, regardless, delivery type and pregnancy had no impact on radiological images in AS patients [26]. However, our study found radiographic differences in AS patients depending on the type of delivery. Using statistical shape analysis, we detected noticeable enlargement in the SIJ in AS patients delivered with V/D.

Nevertheless, none of those studies evaluate the morphometric properties of the SIJ in AS patients regarding the type of delivery. Moreover, our results demonstrated that birth type could affect the morphometric properties of the SIJ even in healthy individuals. Vaginal delivery caused more enlargement at the most anterior part of the SIJ. Birth is a complex set of events in which many different factors come together, and this enlargement in the V/D group could be explained by hormonal mechanisms during those events. Increased laxity of ligamentous structures during pregnancy leads to expansion of the pelvic girdle and loosening of the SIJ, as well as an increase in the amount of synovial fluid in the SIJ [44].

Particularly, vaginal birth, for both healthy and AS individuals, may have adverse effects on the SIJ. The present study results demonstrated that SIJ tended to be enlarged in AS patients in the V/D delivered group, especially on the right side. The study revealed statistical differences in the shape of the SIJ between the types of delivery in patients with AS and found visible expansion and deformation on morphometric images of AS patients delivered with V/D. Even in healthy women who delivered by V/D, statistical shape analysis found enlargement in SIJ compared to healthy C/S-delivered patients.

Significant differences were observed not only in the AS patients between the two types of delivery, but our study showed a dramatic increase and deformation of surface area in SIJ in V/D delivered AS patients, compared to the C/S control group. The present study revealed that in both groups, with AS and control, vaginal-delivered patients showed more pronounced morphometric changes in the right SIJ. The differences, especially for the right SIJ morphometry in both study and control groups, could not be explained but should be evaluated in future studies. As is known, a C/S is a life-saving intervention with fetal and maternal needs, most commonly for obstructed labor with cephalopelvic disproportion indication. Pavličev M et al., in their study, questioned, “Why can evolution not completely eliminate cephalopelvic disproportion?”. The authors emphasized that the human pelvis represents an evolutionary compromise between the function of bipedal gait, better support for visceral organs, and childbirth [45]. In their article, the authors question the evolution of pelvic morphology and examine the adaptive mechanisms of the female pelvis. However, many changes in the human pelvis and SIJ cannot be explained even from an evolutionary perspective, but we believe that the present study’s findings offer opportunities for future research.

### Limitation

This study has certain limitations that should be addressed in future research. First, the radiological evaluations were conducted after delivery, with varying time intervals across the cohort. This variability, inherent to the retrospective design, may have influenced the sacroiliac joint (SIJ) morphology assessment. Second, disease-specific parameters such as the Bath Ankylosing Spondylitis Disease Activity Index (BASDAI) were not evaluated, limiting our ability to correlate disease progression with morphometric changes. Furthermore, since this study was designed retrospectively, the time between the last delivery and X-ray imaging could not be evaluated. Finally, while obstetric history was comprehensively analyzed, other factors, such as hormonal influences during pregnancy and postpartum biomechanical changes, were not explored. Future studies should employ prospective designs with standardized evaluation timelines, detailed disease staging, and broader clinical variables to provide more robust insights into the interplay between delivery mode and SIJ morphology.

## 5. Conclusions

This study provides the first morphometric analysis of sacroiliac joint (SIJ) shape differences in patients with ankylosing spondylitis (AS) and healthy controls based on delivery type. Our findings highlight that vaginal delivery is associated with increased SIJ enlargement in both healthy individuals and those with AS, suggesting that delivery type independently influences joint morphology. The result of the present study advocates that C/S birth may be the protective procedure for SIJ in AS patients. Additionally, these results emphasize the potential need for individualized obstetric planning in AS patients.

Despite its limitations, including the variability in radiological evaluation timing, this study lays a foundation for future research. Prospective studies incorporating disease activity indices, standardized imaging timelines, and broader clinical factors are essential to better understand the delivery mode’s clinical and therapeutic implications in AS patients. Future research can guide more precise management strategies for obstetric and rheumatological care by addressing these gaps.

## Figures and Tables

**Table 1:** Definitions of landmarks used in the present study.

Landmark Number	Description
Landmark 1	Superior tip of iliac crest at the SIJ
Landmark 2	The most anterior part of SIJ on the iliac side
Landmark 3	Middle point of SIJ on the iliac side
Landmark 4	Upper 1/3 of the inferior half of SIJ on the iliac side
Landmark 5	Lower 1/3 of the inferior half of SIJ on the iliac side
Landmark 6	The most inferior point of the SIJ on the iliac side
Landmark 7	The most inferior point of the SIJ on the sacral side
Landmark 8	Lower 1/3 of the inferior half of the SIJ on the sacral side
Landmark 9	Upper 1/3 of the inferior half of the SIJ on the sacral side
Landmark 10	Middle point of the SIJ on the sacral side
Landmark 11	The most anterior part of the SIJ on the sacral side
Landmark 12	Superior tip of the SIJ on the sacral side

**Table 2:** Comparison of age and obstetric data between groups.

	Cases (n = 36)	Controls (n = 34)	*p*-Value
Age (Years)	46.75 ± 9.34	38.71 ± 9.64	<0.001 ^a^
Type of Birth			
V/D	17 (47.20%)	19 (55.90%)	0.469 ^b^
C/S	19 (52.80%)	15 (44.10%)	
Gravida	2.50 (1–8)	2 (1–9)	0.218 ^c^
Parity	2 (1–4)	2 (1–8)	0.270 ^c^
Abortus	10 (27.80%)	8 (23.50%)	0.684 ^b^

Data were presented as mean ± st. deviation, median (minimum–maximum), and n%. ^a^: Independent Samples *t*-Test, ^b^: Chi-square Test, ^c^: Mann–Whitney U Test.

**Table 3:** Distribution of grading of radiographic criteria for Modified New York Stages of right and left sacroiliac joints (SIJ) in ankylosing spondylitis (AS) patients according to delivery type.

Modified New York Criteria		Delivery Type of AS Patients		*p*-Value ^d^
		C/S (n = 19)	V/D (n = 17)	
Right SIJ	Stage I	0	0	0.862
	Stage II	9(47.40%)	7(41.20%)	
	Stage III	9(47.40%)	10(58.80%)	
	Stage IV	1(5.30%)	0	
Left SIJ	Stage I	0	2(11.80%)	0.413
	Stage II	11(57.90%)	9(52.90%)	
	Stage III	6(31.60%)	6(35.30%)	
	Stage IV	2(10.50%)	0	

SIJ: Sacroiliac joint, ^d^: Fisher–Freeman–Halton Test.

## Data Availability

Data are available on request from the corresponding author.
